# Assessment of exposure to soils contaminated with lead, cadmium, and arsenic near a zinc smelter, Cassiopée Study, France, 2008

**DOI:** 10.1007/s10661-015-4587-2

**Published:** 2015-05-14

**Authors:** Cécile Durand, Nicolas Sauthier, Valérie Schwoebel

**Affiliations:** French Institute for Public Health Surveillance (InVS) – Regional Office “Cire Midi-Pyrénées”, Cire Midi-Pyrénées, 10 chemin du raisin, 31050 Toulouse cedex, France

**Keywords:** Exposure, Impregnation, Cadmium, Polluted soils, Screening

## Abstract

After 150 years of industrial activity, significant pollution of surface soils in private gardens and locally produced vegetables with lead, cadmium, and arsenic has recently been observed in Viviez (Southern France). A public health intervention was conducted in 2008 to identify individual health risks of Viviez inhabitants and to analyze their environmental exposure to these pollutants. Children and pregnant women in Viviez were screened for lead poisoning. Urinary cadmium testing was proposed to all inhabitants. Those with urinary cadmium levels over 1 μg/g creatinine were then tested for kidney damage. Urinary cadmium and arsenic levels were compared between participants with non-occupational exposure from Viviez and Montbazens, a nearby town not exposed to these two pollutants, in order to identify environmental factors contributing to impregnation. No case of lead poisoning was detected in Viviez, but 23 % of adults had urinary cadmium over 1 μg/g creatinine, 14 % of whom having markers of kidney damage. Viviez adults had higher levels of urinary cadmium, and to a lesser extent, higher levels of urinary arsenic than those from Montbazens. Consumption of local produce (vegetables and animals) and length of residence in Viviez were associated with higher urinary cadmium levels, independently of known confounding factors, suggesting persisting environmental exposure to contaminated soil. To conclude, health risks related to cadmium exposure were identified in the Viviez population living on contaminated soils. Lead and arsenic exposure did not pose health concerns. Interventions were proposed to reduce exposure and limit health consequences.

## Introduction

### General background

In recent years, concerns about soil conditions in France and their health impact have increased. Health authorities are regularly questioned about health risks and the level of remediation required to rehabilitate former industrial areas where soil is polluted by heavy metals or organic pollutants. By measuring pollutant concentrations in the environment, quantitative health risk assessments can be performed, taking into account all potential ways and sources of exposure to pollutants in different scenarios (WHO 1999). However, because of insufficient knowledge about interindividual variability and pollutant transfer from the environment to humans, these predictions do not always reflect real-world situations. Directly measuring exposure with biological markers can sometimes be useful to evaluate exposure when appropriate and reliable biomarkers exist (Dor et al. 2008, 2012).

### Local context

Viviez, a town in South-Western France, has seen 150 years of industrial activity, zinc smelting being its biggest industry. In 2006, an application to rehabilitate the existing industrial site led to the involvement of the health authorities, as the soil in part of the industrial area was polluted by different heavy metals. In this context, the French Institute for Public Health Surveillance was asked to assess the health impact of the pollution on the population of Viviez and to determine the relevance of conducting health studies. Environmental measures performed in 2006–2007 and analyses of various industrial processes showed that this industrial activity had generated atmospheric emissions (until 1987) and substantial soil pollution, in particular by cadmium, but also by arsenic and lead, with median concentrations in surface soil of private gardens of 27, 140, and 450 mg/kg, approximately 20, 3, and 5 times the reference values, respectively, in a nearby unexposed town. This pollution concerned the whole town of Viviez, particularly the most builtup area in the center near the smelter. Moreover, pollutant concentrations in vegetables grown in the town were high, particularly in root vegetables (for cadmium) and leafy vegetables (for arsenic and cadmium), respectively reaching 1.14 and 1.65 mg/kg (11 and 8 times the European safety limits, respectively). Moderate contamination of private wells was also discovered. Local streams were heavily contaminated, especially by cadmium. Pollution also contaminated the Lot River and in turn the Gironde estuary (more than 200 km away), resulting in the regular contamination of its oysters (Lanceleur et al. 2011). Tap water was uncontaminated as Viviez’s town water supply originates upstream of the source of pollution. Given all these factors, the following sources of exposure had to be taken into account to assess inhabitants’ exposure: ingestion of soil, ingestion, and inhalation of dust and ingestion of locally grown produce.

The main risks of human exposure to lead, cadmium, and arsenic have been extensively described as follows:

- Lead: lead poisoning with neurologic damage and development problems in young children (ATSDR 2007a).
- Cadmium: kidney damage (renal tubular damage often associated with glomerular damage). At the beginning, this damage is reversible but it can develop into kidney failure (ATSDR 2012; Järup et al. 1998).
- Arsenic: skin lesions, cardiovascular problems, and various cancers (ATSDR 2007b).

In the context of Viviez, no particular health problem in the general population without occupational exposure had ever been reported to the authorities. Furthermore, no request regarding this pollution had ever come from the population, essentially composed of sedentary working-class families and elderly people, particularly attached to the industrial past of the town.

### Assessment process

First, a quantitative health risk assessment conducted in 2007–2008 concluded that exposure to lead, cadmium, and arsenic median concentrations in Viviez may have had a health impact on children, adults, and the elderly (Durand et al. 2011; Schwoebel et al. 2013). Consumption of locally grown produce resulted in a clear increase in all calculated risks. However, this assessment was based on exposure hypotheses which were difficult to verify.

Second, we directly investigated the real-world health effects on the population (by contacting health professionals, examining kidney failure registers and the mandatory reporting of lead poisoning). However, the population size was too small and the potential health problems not specific enough to exposure to be able to identify abnormal increases of particular diseases (Durand et al. 2011).

Finally, two different interventions with distinct objectives were implemented as follows:

- Screening for lead poisoning and kidney damage among inhabitants with excess cadmium body burden, the objective being to identify people exceeding an established medical threshold and formulate individual medical recommendations
- Exposure studies to measure impregnation to cadmium and arsenic in order to identify potential excess exposure of Viviez’s inhabitants and to explain exposure factors which increase the body burden of these two pollutants in this population. These studies aimed to formulate recommendations to protect Viviez’s population as a whole.

## Materials and methods

Screening and exposure studies were performed simultaneously in September–October 2008, with a shared protocol and shared financing. However, different populations were concerned.

### Lead poisoning screening

The target population was children aged between 6 months and 7 years and pregnant women, living in Viviez for at least 6 months. We chose this minimum length of residence to ensure that the potential effect of environmental exposure on increased body burden was measurable. The blood lead level (BLL, in μg/l) was measured, reflecting the balance between current exposure, lead stored in bones and released into the blood, and elimination. Above the regulatory threshold of 100 μg/l (article L1334-1 French code of public health), health authorities had to investigate the homes of individuals for sources of poisoning.

### Screening for kidney damage

This screening was performed in two stages. First, urinary cadmium concentration was measured among all volunteer inhabitants over 2 years old, living in Viviez for at least 6 months and without urinary incontinence. This biomarker was chosen because it reflects chronic exposure and the cadmium body burden. All biomarkers were measured by a laboratory selected through public procurement. The call for tenders stipulated a high degree of performance and international quality control certification. The limit of detection (LOD) was 0.015 μg/l and the limit of quantification (LOQ) was 0.044 μg/l.

Many studies have been published on the urinary cadmium threshold beyond which an excess of kidney damage was observed. Results sometimes differ, with the thresholds in more recent studies tending to be lower (2.5 μg/g in JECFA (2003), 2 μg/g in CSTEE (2004), 1 μg/g in Järup et al. (2000), 0.67 μg/g for tubular damage and 0.80 μg/g for glomerular damage in Akesson et al. (2005), 0.5 μg/g by EFSA (2009), and 0.3 μg/g in Thomas et al. (2009)). In accordance with the results of an American study (CDC 2009), we chose a threshold of 1 μg/g creatinine to define an impregnation higher than that in the general population. The cadmium body burden was considered excessive beyond 2 μg/g creatinine (threshold for surveillance of kidney damage used in occupational medicine in France at the time of the study (INRS 2010)). For children, the chosen threshold was 1 μg/g creatinine, which matched the results of a multicenter European study (De Burbure et al. 2006).

In the second stage, kidney damage biomarkers were measured in the same urine sample (first morning urine) in all participants with a urinary cadmium concentration higher than 1 μg/g. Two biomarkers were measured: urinary retinol-binding protein (RBP), which is a sensitive marker of renal tubular damage, and urinary albumin, which reflects the level of renal glomerular filtration. For RBP, the LOD and LOQ were 10 and 50 μg/l, respectively. For albumin, quantification was possible above 11.2 mg/l. Kidney damage was defined as having RBP higher than 300 μg/g creatinine (value used in occupational medicine in France at the time of the study (INRS 2010)) or urinary albumin higher than 2 mg/mmol creatinine (Anaes 2002).

All participants with a urinary cadmium concentration higher than 1 μg/g were requested to consult their doctor in order to assess potential individual sources of exposure. Furthermore, participants with kidney damage were advised to consult a nephrologist.

### Exposure studies

Exposure studies to cadmium and arsenic were cross-sectional. Participants from Viviez were compared with participants living in the unexposed town of Montbazens, chosen for its similarities with Viviez (similar size and structure of population, housing characteristics, occupational, and social characteristics) and its proximity (12 km). Environmental measures confirmed that Montbazens was indeed unexposed to the pollutants studied (median in soils 80, 48, and 1.25 mg/kg for lead, arsenic, and cadmium, respectively). In Viviez, all inhabitants agreeing to participate in the screening for kidney damage also agreed to participate in the exposure studies. Accordingly, inclusion criteria were the same as those mentioned in “Screening for kidney damage” above. However, at the time of analysis, we restricted the exposure studies to participants without occupational exposure. In Montbazens, volunteers had to meet similar inclusion criteria: more than two years old, living in Montbazens for at least 6 months, no urinary incontinence and no occupational exposure. Additionally, they could not have consumed produce grown in Viviez, and not lived, worked or spent any substantial amount of time in Viviez.

For the cadmium exposure study, urinary cadmium was also the biomarker used. For the arsenic exposure study, the biomarker used was urinary arsenic (inorganic arsenic + monomethylarsonic acid (MMA) + dimethylarsenic acid (DMA)), reflecting exposure in recent days. LOD was 1 μg/l and LOQ 3 μg/l. A urinary arsenic threshold of 15 μg/g creatinine was chosen to define an impregnation level higher than that in the general population (Becker et al. 2003).

A face-to-face interview at participants’ homes using a standardized questionnaire collected the following information:

- Individual characteristics: sociodemographic and health information (age, gender, occupational activity, family situation, educational level, body mass index, medical history)
- Pollutant exposure factors unrelated to the area of residence and cited in scientific literature (active and passive smoking, consumption of seafood and offal, consumption of alcohol and mineral water, free time or professional activities where exposure to pollutants was possible, including contact with metal handles or the use of paint and dyes) (ATSDR 2007b, 2012; Fréry et al. 2011)
- Environmental exposure factors related to area of residence (length of residence, consumption of locally grown produce, drinking local well and spring water, frequency of mopping the floor, presence of a private garden in the house, frequency of certain activities in the area like gardening and hunting, etc.)

“Lifetime exposure” and “exposure in recent days” were used, respectively, to assess exposure in the cadmium and arsenic studies.

To analyze levels of urinary cadmium and arsenic, multivariate regressions were conducted separately on adults (over 15 years) and children (2–14 years old) taking into account individual and exposure factors. For the cadmium study, we used linear regression. For the arsenic study, we used the following two methods in order to account for the high proportions of values below LOD and LOQ and to be more confident in the results: Tobit regression for left and interval censored data to explain the variation in the urinary arsenic mean, and logistic regression to measure the risk of having an extreme urinary arsenic level (≥5 μg/l for adults and ≥3 μg/l for children) (Helsel 1990; Lubin et al. 2004).

The alpha risk used was 0.05. All statistical analyses were performed with Stata 11® software.

### Ethics

The French committee for the protection of persons and the French health products safety agency approved the protocol. Inhabitants were free to participate in the study after having been informed individually (mail) and collectively (meetings). Participants signed a consent form (in the case of minors both parents had to sign). Data were captured and analyzed after anonymization. The link between identifiers and names was destroyed after individual screening results were returned.

## Results

### Lead poisoning screening

Among 92 children identified in Viviez, 14 participated in lead poisoning screening (15.2 %). One pregnant woman also participated.

All the BLL measured were between 10 and 35 μg/l. No lead poisoning case was detected. The geometric mean among children was 17.8 μg/l, and the median and the arithmetic mean were both 19 μg/l.

### Screening for kidney damage

Among the 1499 people in the target population, 692 (46.2 %) were screened for kidney damage, including 96 children (50.3 %) and 596 adults (45.6 %). Participants were not significantly different from Viviez’s general population in terms of gender, age, professional activity, length of residence, and type of home.

Among the 596 adults, 136 (22.8 %; 95 % CI 19.4–26.2) had a urinary cadmium concentration higher than 1 μg/g and were thus tested for kidney damage. Thirty (5.0 %; 95 % CI 3.3–6.8) of the latter had excessive impregnation (≥2 μg/g).

These 136 people were mostly older adults (71 % over 60 years), females (66 %) and living in Viviez for over 20 years (89 %).

**Fig. 1:**
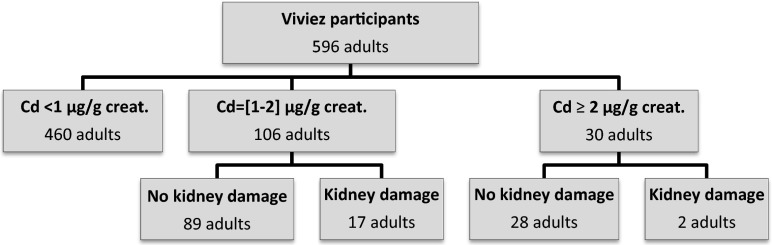
Results of screening of kidney damage according to urinary cadmium concentration among participants from Viviez, Cassiopée study, 2008

Among them, 19 people with kidney damage were identified (14.0 %; 95 % CI 8.1–19.9), of whom 11 had an RBP level above the threshold and 14 excessive albumin excretion. The profile of these 19 people was not different from that of the 117 without kidney damage (Fig. 1).

Among the 96 participating children, one (1.0 %; 95 % CI 0.0–3.1) had excessive cadmium impregnation (≥1 μg/g) but no kidney damage.

### Exposure studies

#### Adult exposure to cadmium

The study included 385 adults in Viviez and 290 adults in Montbazens, with no current or past occupational exposure to cadmium.

In Viviez, 21.6 % of adult participants had cadmium levels higher than 1 μg/g and 4.9 % had levels higher than 2 μg/g. This corresponded to 3.8 and 0 %, respectively, in Montbazens. The unadjusted geometric mean (GM) of urinary cadmium in Viviez (0.49 μg/g; 95 % CI 0.45–0.54) was higher than that in Montbazens (0.31 μg/g; 95 % CI 0.29–0.34) (*p* < 10^−3^).

Several individual characteristics and exposure factors unrelated to the area of residence were associated with urinary cadmium in both towns:

- Gender: the urinary cadmium mean was higher in women than in men (*p* < 10^−3^)
- Age: urinary cadmium increased with age, following a cubic relationship (increase stabilized at older ages) (*p* < 10^−3^)
- Creatinine (representing the dilution of urine): urinary cadmium (in μg/l) increased linearly with creatinine (in g/l) (*p* < 10^−3^)
- Educational level and occupational activity: the urinary cadmium mean was lower in people with higher education levels (*p* = 0.024) and in the nonworking population (*p* = 0.025)
- Smoking: the urinary cadmium mean was higher in smokers, increasing linearly with tobacco consumption (+5.9 % of urinary cadmium per 100 gram-year of tobacco; 95 % CI 4.1–7.8 %; *p* < 10^−3^). Similarly, the urinary cadmium mean was higher in current smokers (0.52 μg/g; 95 % CI 0.47–0.57) than in former smokers (0.43 μg/g; 95 % CI 0.39–0.47) and in nonsmokers (0.39 μg/g; 95 % CI 0.37–0.41). It was also higher in people exposed to passive smoking (*p* = 0.007)

In Viviez, three environmental exposure factors were associated with urinary cadmium whereas none was associated in Montbazens (Table 1):

**Table 1:** Urinary cadmium adjusted GM^*^ or variation of adjusted GM in adults included in the exposure study, according to environmental exposure factors, Cassiopée study, 2008

	Viviez (*N* = 375)		Montbazens (*N* = 282)	
	% of variation of GM (95 % CI)	*p*	% of variation of GM (95 % CI)	*p*
Length of residence				
By 1-year increase	1.2 (0.8, 1.5)	<10^−3^	−0.3 (−0.7, 0.03)	0.071
By 5-year increase	5.9 (4.3, 7.5)		−1.6 (−3.2, 0.1)	
By 10-year increase	12.2 (8.8, 15.7)		−3.1 (−6.4, 0.3)	
	GM (95 % CI)	*p*	GM (95 % CI)	*p*
Part of consumption of home-grown fruits and vegetables				
Less than 10 %	0.48 (0.44, 0.51)	0.008	0.32 (0.29, 0.35)	0.392
Approximately 25 %	0.53 (0.44, 0.63)		0.34 (0.29, 0.40)	
Approximately 50 %	0.56 (0.45, 0.70)		0.38 (0.32, 0.44)	
75 % and over	0.77 (0.59, 1.02)		0.33 (0.28, 0.39)	
Consumption of home-grown animal produce (eggs, poultry, rabbits)				
No	0.48 (0.45, 0.51)	0.005	0.33 (0.30, 0.36)	0.649
Yes	0.63 (0.53, 0.76)		0.34 (0.31, 0.37)	

^*^Geometric mean (GM) (μg/g creatinine) adjusted for creatinine, gender, age, educational level, professional activity, smoking, consumption of seafood and offal

- Local fruit and vegetables consumption: the urinary cadmium mean was higher in Viviez inhabitants for whom locally grown produce constituted a large proportion of their fruit and vegetables consumption, and particularly in people who grew 75 % or more of the fruit and vegetables they ate (*p* = 0.008).
- Consumption of local animal produce: the urinary cadmium mean was higher in Viviez participants who consumed local animal produce (eggs, poultry, and rabbits) (*p* = 0.005).
- Length of residence: the urinary cadmium mean increased linearly (1.2 % per year) with length of residence for people living in Viviez (*p* < 10^−3^). This association was still found when the analysis was restricted to people who did not consume locally grown produce.

These three factors explained 8 % of the cadmium body burden variability in Viviez’s population, as opposed to the 0 % in Montbazens’s population. After adjustment for all factors, the difference in geometric mean urinary cadmium between the exposed (0.51 μg/g; 95 % CI 0.48–0.55) and unexposed towns (0.33 μg/g; 95 % CI 0.31–0.35) was still significant (*p* < 10^−3^).

To improve assessment of current exposure, the analysis was restricted to adults living in their respective town for less than 20 years (after the cessation of atmospheric emissions from the Viviez smelter). The same environmental exposure factors were associated with urinary cadmium in Viviez (length of residence *p* < 10^−3^, local fruit and vegetables consumption *p* = 0.011, local animal produce consumption *p* = 0.053) but not in Montbazens. The adjusted geometric mean in Viviez (0.30 μg/g; 95 % CI 0.27–0.33) was slightly higher than in Montbazens (0.26 μg/g; 95 % CI 0.24–0.29), but this difference was of borderline significance (*p* = 0.060).

#### Adult exposure to arsenic

The study included 518 adults in Viviez and 290 adults in Montbazens with no current occupational exposure to arsenic.

Four adults (0.8 %) in Viviez and two (0.7 %) in Montbazens had an arsenic impregnation level higher than the threshold of 15 μg/g creatinine. Furthermore, the urinary arsenic level was lower than the LOD in 35 % of adults (34 % in Viviez and 38 % in Montbazens) and between the LOD and the LOQ in another 35 % (37 % in Viviez and 33 % in Montbazens). Beyond these limits of quantification, the 75th percentile was equal in both towns (3.4 μg/l) but the 95th percentile was higher in Viviez (9.2 μg/l) than in Montbazens (7.4 μg/l).

The following sociodemographic and exposure factors unrelated to the area of residence were associated with increased urinary arsenic mean, both in Viviez and Montbazens: older age (*p* = 0.017), female gender (*p* = 0.031 in Viviez only), creatinine (*p* < 10^−3^), normal or low body mass index (*p* = 0.002), recent consumption of seafood (*p* = 0.014) and wine (*p* = 0.005 in Viviez only), and current active and passive smoking (*p* = 0.004). In the second analytical method used (logistic regression), these same factors also explained the risk of having an arsenic impregnation level higher than 5 μg/l.

Several environmental exposure factors were associated with increased urinary arsenic mean in Viviez but not in Montbazens: recent consumption of locally-raised poultry (*p* = 0.049), consumption of local well and spring water (*p* = 0.001), low frequency of mopping the floor (*p* = 0.028), and frequency of gardening (*p* = 0.029). The first two factors were also significant in the logistic regression model.

Finally, the final Tobit regression model concluded that the urinary arsenic mean was higher for adults without occupational exposure living in Viviez than for adults living in Montbazens (+24.5 %; 95 % CI 5.7–46.6, *p* = 0.009) after adjustment for individual characteristics and exposure factors. Moreover, the final logistic regression model, with the same adjustment factors, also concluded that adults in Viviez had a higher risk of having an impregnation above 5 μg/l than their Montbazens counterparts (OR = 2.92, 95 % CI 1.57–5.43, *p* = 0.001). This increased risk was particularly high in nonsmokers (OR = 7.51, 95 % CI 2.60–21.70; *p* < 10^−3^). Overall, the two methods used provided similar results regarding exposure factors and the differences in impregnation levels.

#### Child exposure to cadmium

The study included 152 children: 92 in Viviez and 60 in Montbazens.

As was the case for adults, urinary cadmium (in μg/l) in children increased linearly with creatinine (*p* < 10^−3^). It was also associated with age following a cubic relationship (highest impregnation for children approximately two years old, then a decrease followed by a slight increase for teenagers approximately 14 years old) (*p* < 10^−3^).

Length of residence was associated with urinary cadmium levels in Viviez (higher GM for children who lived in the town for 4–7 years compared with other groups) (*p* < 10^−3^). In addition, the GM was higher in children from Viviez who occasionally or regularly put their hands or objects in their mouth than in those who did not (*p* = 0.011). These two associations were not observed for children from Montbazens.

However, even after taking these factors into account, the adjusted cadmium GM was not different between children living in Viviez (0.14 μg/g; 95 % CI 0.12–0.15) and those living in Montbazens (0.12 μg/g; 95 % CI 0.11–0.14) (*p* = 0.288).

#### Child exposure to arsenic

The urinary arsenic concentration was lower than the LOD in 37 % of the 152 children (29 % in Viviez and 48 % in Montbazens) and between the LOD and the LOQ in 35 % (41 % in Viviez and 25 % in Montbazens).

The urinary arsenic mean was higher in children living in Viviez than in those living in Montbazens (+51.2 %; 95 % CI 5.3–117.3, *p* = 0.025) after adjustment for age, gender, creatinine, parents’ educational level, corpulence, recent consumption of fish, and current exposure to passive smoking. Nevertheless, in the logistic model adjusted for the same factors, the risk of having a urinary arsenic level above 3 μg/l was not higher in children from Viviez than in their Montbazens counterparts (OR = 1.39, 95 % CI 0.55–3.48, *p* = 0.479). No exposure factor related to the area of residence was associated with urinary arsenic in Viviez or Montbazens in the two regression models used.

## Discussion

### Exposure to cadmium

Our study showed that adults living on soils heavily contaminated with cadmium in Viviez had a higher impregnation to cadmium than both a neighbouring population living on uncontaminated soils and the general population in France and in countries with similar dietary environments.

The 22.8 % of adult participants with had a urinary cadmium concentration higher than 1 μg/g and were requested to consult their doctor. Of these, 5.0 % had an excessive impregnation level (≥2 μg/g). After exclusion of adults exposed to cadmium at work, these percentages were respectively 21.6 and 4.9 % in Viviez, and only 3.8 and 0 % in Montbazens. A recent study on French general population showed that 0.3 % of participants between 18 and 74 years had urinary cadmium exceeding 2 μg/g, with a geometric mean of 0.29 μg/g (Fréry et al. 2011). This was much lower than the 3.5 % and 0.47 μg/g observed in Viviez in the same age group. In comparison with other countries, the urinary cadmium mean in Viviez adults was higher than that observed in representative samples of the United States population in the Nhanes study (0.27 μg/g) (CDC 2009), in Canada (0.35 μg/g) (Santé Canada 2010), in the Czech Republic (0.24 μg/g) (NIPH 2010), and in Germany (0.23 μg/g) (Becker et al. 2003). It was however lower than those observed in the Chinese (1.83 μg/g) (Jin et al. 2004) and Japanese populations (1.3 μg/g in women over 35 years) (Ezaki et al. 2003), but environment and eating habits are different in those countries (e.g., high contamination of rice by cadmium).

Screening for kidney damage identified 19 adults (14.0 %) with an excess of RBP (8.1 %) or albumin (10.3 %) among the 136 adults with an excessive cadmium body burden. Comparison of this percentage with the results of other studies is difficult because biomarkers of kidney damage were measured only in some participants following their urinary cadmium results, and so, prevalence of kidney damage in the total population of Viviez could not be estimated. However, the percentage of renal tubular damage (measured by RBP) in this subpopulation was slightly higher than that observed in the general population in Sweden (8.1 vs 5 %) (Järup et al. 2000).

Most of the sociodemographic and exposure factors unrelated to the area which were associated with urinary cadmium (age, gender, creatinine, smoking, educational level, and occupational activity) have already been described in the literature (Conrad et al. 2010; Fréry et al. 2009, 2011; Levy et al. 2007; Moriguchi et al. 2005; Olsson et al. 2002; Richter et al. 2009; Ruiz et al. 2010; Sartor et al. 1992). In particular, the increase in urinary cadmium with age followed by stabilization in older ages has been documented (Fréry et al. 2011; Moriguchi et al. 2005). Moreover, the linear increase of urinary cadmium concentrations with tobacco consumption is consistent with the well-known influence of smoking on cadmium exposure (Conrad et al. 2010; Fréry et al. 2011; Levy et al. 2007; Olsson et al. 2002; Richter et al. 2009).

The association of urinary cadmium in the exposed adult population with three factors related to the area of residence (length of residence, consumption of local fruit and vegetables, and consumption of local animal produce), supports the hypothesis that inhabitants from Viviez had higher cadmium impregnation than unexposed inhabitants from a neighbouring town because of environmental exposure.

High cadmium concentrations found in vegetables and results on consumption of locally grown vegetables suggest exposure by ingestion. Indeed, a dose-response relationship was observed between the proportion of locally grown vegetables consumed and the urinary cadmium mean, which reinforces the plausibility of the result. Consumption of home-grown vegetables was also found to be a possible cadmium exposure pathway in a recent Swedish study (Hellström et al. 2007).

The linear increase in urinary cadmium with increasing length of residence may reflect cumulative exposure by ingestion (because of the cumulative length of consumption of locally grown produce), by inhalation or ingestion of dust, or by inhalation of atmospheric emissions generated before 1987. Exposure exclusively by ingestion is unlikely, because the length of residence was still associated with urinary cadmium, and a difference between both towns was still observed when the analysis was restricted to people who did not consume locally grown produce. The influence of dust (indoors and outdoors) and of atmospheric emissions on urinary cadmium has been demonstrated in various studies conducted near zinc smelters (Hogervorst et al. 2007; Thomas et al. 2009). The results found on adults who had moved to Viviez more recently (i.e., after the cessation of atmospheric emissions) and the current environmental context suggest that past atmospheric emissions cannot be an exclusive source of exposure and that exposure by inhalation or ingestion of dust still persists.

Although it is difficult to establish a temporal relationship between exposure and body burden in such a cross-sectional study, these results nevertheless suggest current environmental exposure to cadmium for nonoccupationally exposed adults from Viviez.

Among children, only 1 % of participants had excessive cadmium impregnation (≥1 μg/g). No difference in urinary cadmium mean was found between children from Viviez and from Montbazens. There is no study in the French children population which we can compare our results with. The urinary cadmium means observed in Viviez and in Montbazens (respectively 0.13 and 0.12 μg/g in 6–11 years) were close to those observed in the Nhanes study in children from the USA (0.09 μg/g in 6–11 years) (CDC 2009). However, even though the cadmium mean was not different between the exposed and unexposed towns, the association between the habit of putting one’s hands to one’s mouth and increased urinary cadmium also suggests exposure by ingesting dust.

### Exposure to arsenic

Slight differences in levels of urinary arsenic were found between adults from Viviez and from Montbazens, and certain environmental exposure factors were associated with urinary arsenic in Viviez but not in Montbazens. This suggests that the environment in Viviez may have played a role in arsenic exposure and that the most likely exposure was through inhaling or ingesting contaminated dust.

However, because of the large proportion of censored data, the urinary arsenic mean was difficult to estimate precisely and results should be interpreted with caution. Only the 75th percentiles and over were reliable because they were above the detection limit. According to these percentiles, levels of urinary arsenic among adults in Viviez and Montbazens who were not occupationally exposed to arsenic were lower than or similar to levels observed in other studies in the general population in France and other countries. The 95th percentile among adults between 18 and 74 years in France was 10.7 μg/l and 75th percentile was 6.3 μg/l (Fréry et al. 2011) versus, respectively, 9.2 and 3.6 μg/l in Viviez and 8.2 and 3.6 μg/l in Montbazens. In other countries, 95th percentiles are generally higher than those in France, examples being 15.2 μg/g in Germany (Becker et al. 2003), 12.1 μg/g in the Czech Republic (Spevackova et al. 2002), and 18.9 μg/l in the USA (CDC 2009).

Thus, our results did not suggest current excessive arsenic exposure in the Viviez adult population compared with the French general population or that of other countries.

Among children, the difference in urinary arsenic levels between children from Viviez and Montbazens was not clearly established and no exposure factor related to the area of residence was associated with urinary arsenic in Viviez or in Montbazens.

Thus, arsenic exposure was not a current major health concern in Viviez, although past environmental exposure was very likely and current exposure was still possible at lower levels.

### Exposure to lead

No lead poisoning case was detected in Viviez. However, participation in the screening was low despite the information given to parents. Accordingly, we cannot conclude that there is no lead poisoning problem in children and pregnant women from Viviez.

### Limitations

There are several limitations to our study. The selection of the Viviez study population was based on voluntary participation, which accounted for approximately 50 % of those invited. Participants may have been more motivated to participate because of health problems, or exposure to some potential exposure factors. However, the following elements suggest that any selection bias was minimal:

- Participants were unaware of impregnation at the time of the study. The exclusion of occupationally exposed inhabitants also enabled us to exclude individuals who had already been screened for these pollutants at work.
- These levels of arsenic and cadmium impregnation are subclinical most of the time. Therefore, it would be unlikely that a health problem related to impregnation without clinical signs would greatly influence participation.
- Inhabitants with particular exposure, for example from a high level of consumption of locally grown produce, may have felt more inclined to participate in the study. However, all potential exposure factors previously identified and communicated to residents during information meetings were measured and taken into account in the analysis.
- No differences in sociodemographic characteristics were observed between participants and nonparticipants (according to available population data in the two towns).
- Levels of cadmium impregnation in Montbazens were consistent with those in national studies, which suggest that our estimates were indeed representative.

As with any biological measures, our study had analytical limits (LOD, LOQ). These limits were greater for arsenic than for cadmium. To lessen their impact, two different models for censored data were used. We found good agreement between the results of each model, which reinforces the reliability of our conclusions.

Because of the use of declarative information collected by a questionnaire, recall bias is also possible: inaccuracy of participants’ memory may have influenced answers they provided about certain lifetime exposures. Moreover, some answers (mopping the floor, consumption of soil for children, consumption of alcohol, etc.) may have been influenced by the fear that the investigator would judge them. Such a potential response bias was limited, however, because neither the participants nor the interviewers knew the results of the biomarkers at the time of the interview. Therefore, the answers of those participants with higher impregnation levels were unlikely to be more biased than those of other participants.

The interpretation of a cumulative biomarker such as urinary cadmium is difficult in such a cross-sectional study. Indeed, the temporal relationship between exposure and body burden is impossible to evaluate. Nevertheless, the analysis conducted on a subgroup of those most recently arrived to Viviez provided us with an indication on this temporal relationship and helped us to elaborate conclusions.

Finally, the consistency of the results of our analyses using different models examining two different pollutants, and their consistency with those reported in the literature enhance the reliability of our results.

## Conclusions

This is one of the first French studies to aim to measure and explain the cadmium body burden in a population exposed to industrially polluted soils with such a high level of cadmium in the environment.

The results indicate that environmental exposure to cadmium was substantial in Viviez, while exposure to arsenic did not pose any health concern. The main health risk created by cadmium exposure was the increased risk of kidney damage, which has been defined as being significant above the threshold of 1 μg/g of creatinine in most recent studies.

These results highlighted the need to introduce interventions to reduce exposure and limit health consequences. A medical examination was proposed to Viviez adults who had excessive cadmium impregnation. Several recommendations were made to the population in order to limit exposure including reducing consumption of locally grown produce and limiting exposure to dust by taking hygiene precautions (e.g., washing hands and floors of the house regularly). Moreover, treating the soil of private gardens and public spaces of the town was also recommended in order to stop long-term dust exposure.

## Abbreviations

BLL: Blood lead level
CI: Confidence interval
DMA: Dimethylarsenic acid
GM: Geometric mean
LOD: Limit of detection
LOQ: Limit of quantification
MMA: Monomethylarsenic acid
OR: Odds ratio
RBP: Retinol-binding protein
